# Cortical glia in SOD1(G93A) mice are subtly affected by ALS-like pathology

**DOI:** 10.1038/s41598-023-33608-y

**Published:** 2023-04-21

**Authors:** Tereza Filipi, Zuzana Matusova, Pavel Abaffy, Ondrej Vanatko, Jana Tureckova, Sarka Benesova, Monika Kubiskova, Denisa Kirdajova, Jakub Zahumensky, Lukas Valihrach, Miroslava Anderova

**Affiliations:** 1 Department of Cellular Neurophysiology, Institute of Experimental Medicine, Czech Academy of Sciences, Videnska 1083, 14220 Prague, Czech Republic; 2 Second Faculty of Medicine, Charles University, V Uvalu 84, 15006 Prague, Czech Republic; 3 Laboratory of Gene Expression, Institute of Biotechnology CAS, BIOCEV, Prumyslova 595, 25250 Vestec, Czech Republic; 4 Faculty of Science, Charles University, Albertov 6, 12800 Prague, Czech Republic; 5 Department of Informatics and Chemistry, Faculty of Chemical Technology, University of Chemistry and Technology, Technicka 5, 16628 Prague, Czech Republic; 6 Department of Functional Organization of Biomembranes, Institute of Experimental Medicine, Czech Academy of Sciences, Videnska 1083, 14220 Prague, Czech Republic

**Keywords:** Transcriptomics, Glial biology, Amyotrophic lateral sclerosis, Cellular neuroscience

## Abstract

The role of glia in amyotrophic lateral sclerosis (ALS) is undeniable. Their disease-related activity has been extensively studied in the spinal cord, but only partly in the brain. We present herein a comprehensive study of glia in the cortex of SOD1(G93A) mice—a widely used model of ALS. Using single-cell RNA sequencing (scRNA-seq) and immunohistochemistry, we inspected astrocytes, microglia, and oligodendrocytes, in four stages of the disease, respecting the factor of sex. We report minimal changes of glia throughout the disease progression and regardless of sex. Pseudobulk and single-cell analyses revealed subtle disease-related transcriptional alterations at the end-stage in microglia and oligodendrocytes, which were supported by immunohistochemistry. Therefore, our data support the hypothesis that the SOD1(G93A) mouse cortex does not recapitulate the disease in patients, and we recommend the use of a different model for future studies of the cortical ALS pathology.

## Introduction

The characteristic degeneration of motor neurons (MNs) in ALS initially causes progressive muscle atrophy leading to difficulties with movement, speaking and swallowing, and respiratory failure in the final stage. Generally, ALS is considered as a multifactorial disease with poorly understood pathological mechanisms, and with a median survival of three to five years. There is currently no cure or prevention available, only symptomatic treatment.

Although MNs are recognized as the primary cell type affected by the pathology, multiple studies have confirmed that also non-neuronal cells including glia undergo changes and participate in ALS progression^1,2^. Glial cells respond to neurodegeneration or injury by various mechanisms. Microglia and astrocytes acquire a so-called reactive state marked by changes of gene expression and morphology. By the activation of inflammatory and anti-inflammatory pathways, they protect the tissue from further damage. However, in the chronic stage of diseases they have a harmful effect and contribute to the progression of neurodegeneration. Traditionally, reactive astrocytes and microglia were divided into A1 and A2 or M1 and M2 subtypes, respectively. However, this terminology is currently considered outdated, as many various subpopulations of glia with specific gene signatures have recently been described in different disease models^3–5^. Disease-associated astrocytes (DAA), disease-associated microglia (DAM), activated response microglia (ARM), and interferon response microglia (IRM), are just a few examples of these. Oligodendrocytes, on the other hand, are more susceptible to pathological changes. In reaction to disease, they tend to degenerate, rather than transform into a reactive state. However, their passive role in the progression of disease has recently been questioned by studies describing their contribution to immunoprotection, interferon signaling and antigen processing and presentation^6,7^.

The role of astrocytes, microglia and oligodendrocytes, and their respective pathology-related changes, have been reported in ALS patients multiple times^8–10^. The majority of available data come from the spinal cord, but a few studies also reported glial pathology in the cortex^11^. The known pathological changes were mostly identified in *post mortem* tissue, which does not allow for study of the disease mechanisms, increasing the need for a reliable animal model. Currently, the SOD1(G93A) mouse represents the most widely used model resembling familial ALS^12^. Phenotypically the model matches the disease course, and studies in the spinal cord and brainstem reported ALS-related cellular changes previously found in patients^2,8,13–16^. The cortex, however, seems to be a subject of controversy. The number of studies is limited, and the results suggest contradictory outcomes. While some show that cortical glial cells and MNs are affected by the ALS-like phenotype^17–20^, others report no effect in the cortical area in the SOD1(G93A) model^21^.

In this study, we aimed to provide a comprehensive insight into the SOD1 glial pathology in the cortex of the SOD1(G93A) mouse. Combining robust and high-throughput methods such as scRNA-seq and immunohistochemistry, we analyzed astrocytes, microglia, and oligodendrocytes, during the complete course of the disease, which was characterized by behavioral tests. The scRNA-seq was used to monitor transcriptional changes in individual glial cells in time, and to investigate the composition of distinct glial populations with a particular focus on disease-associated subpopulations. To complete the characterization of the cortical pathology, we evaluated the morphological and quantitative changes of glial cells using immunohistochemistry, measuring canonical protein markers.

## Methods

### Animals

For all experiments, we used transgenic mice expressing high levels of human SOD1(G93A) (JAX Strain: 004435 C57BL/6 J-Tg (SOD1*G93A)1Gur/J) and their non-carrier littermates^12^. This strain contains ~ 25 copies of the transgene, and its 50% survival ranges 157 ± 9.3 days (https://www.jax.org/strain/004435). All experimental protocols were approved by the Czech Republic Animal Care Commitee (approval number 40/2019). All methods using animals were carried out in accordance with the European Communities Council Directive (86/609/EEC). All animals used for experiments were sacrificed using pentobarbital followed by decapitation. Due to an advanced stage of the disease, mutant mice were euthanized using carbon dioxide shortly after reaching five months of age. All efforts were made to minimize both the suffering and the number of animals used. The study is reported in accordance with the ARRIVE guidelines.

### Behavioral testing

We conducted the wire grid hang test and the rota-rod test (Mouse RotaRod NG 47650, Ugo Basile, Italy) to assess muscle strength, function, and coordination throughout the disease. Weight was also measured as an additional parameter of the symptom progression. Testing consisted of a single three-attempt session every week, beginning at P30, and lasted for 14 weeks. Before the experiment, all animals performed training. Data are presented as mean or mean ± standard error of the mean (SEM) for n animals. Repeated measures two-way ANOVA with Holm-Sidak’s multiple comparison correction was used to analyze the differences between groups.

### Wire grid hang test

The mouse was placed on a custom-made wire lid, approximately 60 cm above a wood chip covered bottom, and turned upside down. The latency to fall was measured. At the beginning of a testing period, we trained each mouse three consecutive times for at least 180 s. In the experimental session, the mouse had three attempts to hold on to the lid. We noted the best score out of the three with a maximum of 180 s.

### Rota-rod test

The mouse was placed on a stationary rod facing against the direction of rotation. The rod started rotating at a constant speed of 15 rpm, and the latency to fall was measured. Each mouse was trained three consecutive times of at least 180 s at 5, 10 and 15 rpm speed. In the experimental session, the mouse had three attempts to remain on the rod. We noted the best score out of the three with a maximum of 180 s.

### Immunohistochemistry

For immunohistochemical analyses, the animals were deeply anesthetized with PTB (100 mg/kg, i.p.), perfused transcardially with 20 ml of saline solution followed by 20 ml of cooled 4% paraformaldehyde (PFA) in 0.1 M phosphate buffer and decapitated. The brains and spinal cords were dissected out, postfixed overnight with PFA and treated with a sucrose gradient (ranging from 10 to 30%) for cryoprotection. Coronal 30-μm-thick slices were prepared using a cryostat (Leica CM1850, Leica Microsystems, Wetzlar, Germany).

For immunohistochemical staining, the slices were washed in a phosphate buffer saline followed by blocking of the nonspecific binding sites with 5% Chemiblocker (Millipore, Billerica, MA), and 0.2% Triton in phosphate buffer saline. The blocking solution was also used as the diluent for the antisera. The slices were incubated with the primary antibodies overnight, and the secondary antibodies were applied for 2 h at 4–8 °C.

The following primary antibodies were used: rabbit anti-aldehyde dehydrogenase 1 family, member L1 (ALDH1L1 1:500; Abcam, Cambridge, UK), rat anti-myelin basic protein (MBP, 1:500, Biorad, Hercules, CA, US), rabbit anti-choline acetyltransferase (ChAT, 1:200, Merck, Darmstadt, Germany), rabbit anti-ionized calcium-binding adapter molecule 1 (Iba-1, 1:500, Abcam, Cambridge, UK), rabbit cleaved caspase-3 (CC3, 1:50, CellSignaling, Massachusetts, USA) and adenomatous polyposis coli (APC, 1:200, Merck, Darmstadt, Germany). The secondary antibodies were goat anti-rabbit IgG or goat anti-mouse IgG conjugated with Alexa Fluor 488, and chicken anti-rat IgG conjugated with Alexa Fluor 488 (1:500, Invitrogen, Waltham, MA, US). Cell nuclei were visualized by DAPI staining (Merck, Darmstadt, Germany). A Zeiss LSM 880 Airyscan confocal microscope equipped with Ar/HeNe lasers and × 40 water or × 63 oil objectives were used for the immunohistochemical analysis.

### Image analysis and quantification

All analysis were done using FIJI image processing software (ImageJ 2.9.0/1.53t)^22^. Confocal images (212 × 212 × 30 μm) were taken from brain coronal slices (1 mm and 2 mm from bregma), covering area of primary and secondary motor and primary somatosensory cortex (five to six zones per hemisphere).

To quantify the ALDH1L1 fluorescence intensity, we used six animals for each group and two slices from each animal. The thresholding method (Yen method) was used to filter out the background. We calculated the mean integrated density limited to the threshold for each animal.

To quantify changes in morphology of microglia, we conducted Sholl analysis on IBA1 positive cells using Sholl analysis plugin^23^. We used six animals for group and two brain slices from each animal. For each brain slice, a minimum of eight cells was analyzed. For the Sholl analysis, the consecutive z-stack images were converted to maximum intensity projection and the projection was thresholded for creating a binary mask. We counted the number of intersections starting from 5 μm from the center of soma, with radius step size of 5 μm.

To quantify the APC and CC3-positive cells, three animals from each group and one slice (1 mm from bregma) from each animal was used for the analysis. The number of APC or CC3-positive cells was determined from superimposed images and expressed as the percentage of marker expressing cells from the total number of DAPI + cells. The percentage of apoptotic cells was then expressed as a ratio of CC3 + to the previously counted APC + cells.

An Olympus FV10i confocal microscope equipped with × 60 oil objective was used for the analysis of MBP staining. We used six animals for each group and two slices for each animal and scanned 12 zones per each hemisphere. MBP expression density was determined using custom-written FIJI (ImageJ) macro (available at: https://github.com/jakubzahumensky/JT_paper). In brief, to keep the dimensionality of analyzed images equal, the macro extracted a substack of the 20 brightest frames from each z-stack. This was followed by creating a binary mask of the fibers in each frame and measurement of the frame fraction covered by the mask. Within each substack, the mean of this value was calculated, resulting in the volume fraction taken up by the fibers. The statistical analysis of the differences among groups was performed using unpaired t-test. All error bars in plots represent standard error of mean (SEM).

### The preparation of single-cell suspension

The mice were deeply anaesthetized with pentobarbital (PTB) (100 mg/kg, i.p.), and perfused transcardially with a cold (4–8 °C) isolation buffer containing (in mM): NaCl 136.0, KCl 5.4, HEPES 10.0, glucose 5.5, osmolality 290 ± 3 mOsmol/kg. We isolated motor and primary somatosensory cortex and followed the Adult Brain dissociation protocol for mice and rats (Milteyni-Biotec, Germany) but omitted the red blood cell removal step. To prevent the activation of immediate early genes (IEGs), we used transcriptional inhibitor actinomycin D (Sigma–Aldrich, St. Louis, MO), 30 μM during enzymatic dissociation and 3 μM in the following steps^24^. After the debris removal, the cells were layered on top of 5 ml of ovomucoid inhibitor solution (Worthington, NJ) and harvested by centrifugation (300 × *g* for 6 min). Potential cell aggregates were removed by 70 μm cell strainers (Becton Dickinson, NJ). We labeled the final suspension with ACSA-2, Cd11b and O4 antibodies conjugated with allophycocyanin and phycoerythrin respectively (4 °C, 10 min; Miltenyi-Biotec, Germany) to allow for the enrichment of astrocytes^25^, microglia and oligodendrocytes. The cells were enriched using flow cytometry (FACS; BD Influx), calibrated to sort ACSA-2 + , Cd11b + and O4 + cells. Hoechst 33258 (ThermoFisher Scientific, Waltham, MA) was used to check viability. The cells were collected into 200 μl of Advanced Dulbecco's Modified Eagle Medium, supplemented with 10% fetal bovine serum (ThermoFisher Scientific Waltham, MA). Four animals per condition were pooled for the preparation of cell suspension. After FACS, the cell suspension was spun down, concentrated, and used for library preparation.

### scRNA-seq

Chromium Next GEM Single Cell 3' Reagent Kits v3.1 (10 × Genomics, Pleasanton, CA) was used to prepare the sequencing libraries, and the protocol was performed according to the manufacturer’s instructions. Briefly, 10 × Chromium platform was used to encapsulate individual cells into droplets along with beads covered in cell-specific 10 × Barcodes, unique molecular identifiers (UMIs) and poly(dT) sequences. After reverse transcription, the cDNA libraries were amplified (13–14 cycles), fragmented and ligated to sequencing adaptors. SPRISelect magnetic beads were used for purification of the cDNA suspension and size selection of the fragments. Concentration and quality of the libraries was measured using Qubit dsDNA HS Assay Kit (Invitrogen) and Fragment Analyzer HS NGS Fragment Kit (#DNF-474, Agilent). The libraries were pooled and sequenced in paired-end mode using Illumina NovaSeq 6000 SP Reagent Kit, Read 1 containing a barcode and a UMI, and Read 2 covering the sequence of interest. Sequencing data comprised of approx. 100–200 million reads per sample (Supp. Tab. 4).

### Data analysis

The sequencing data were aligned to the reference mouse genome GRCm38 and annotated (GENCODE version M8 annotation) by STARsolo (STAR version 2.7.3a)^26^. EmptyDrops function (DropletUtils R package)^27^ with a threshold of 100 UMIs and FDR <  = 0.001 was applied to preserve only cell-containing droplets. Cells were counted based on the barcodes specific for each droplet/cell. The final number of detected cells differed among samples and was in the range approx. from 2500 to 6800 cells (Supp. Tab. 4).

The data were further processed using Seurat R package (version 4.1.1)^28^. First, data from all samples were SCTransformed and integrated (excluding mitochondrial and ribosomal genes, prefixed by mt- or Rps/Rpl, respectively). Uniform Manifold Approximation and Projection (UMAP) was used to visualize 17 principal components (PC), which were subsequently clustered (FindNeighbors and FindClusters functions, UMAP resolution 0.5). Clusters were annotated based on the expression of known marker genes of the expected cell populations, and their correspondence to the markers found by the FindAllMarkers function (at least 80 % cells in the cluster expressing the markers). DoubletFinder^29^ R package was used for the identification of droplets potentially containing more than one cell. Doublet formation rate was set to 3.9 % as estimated by 10 × Genomics, and the data were processed according to the authors’ recommendations. Clusters expressing ambiguous markers and containing a higher number of doublets were filtered out of the data set.

Sex (male, female) was assigned to individual cells based on the expression of genes encoded by X (*Xist*) and Y chromosome, and those not matching our criteria (male: counts of *Xist* < 1, nFeature_Y > 0; female: counts of *Xist* > 0, nFeature_Y < 2, nCount_Y < 2; nFeature_Y being a number of Y-encoded genes and nCount_Y being a number of transcripts mapping to Y chromosome) were excluded from the dataset (Supp. Fig. 1a). These cells classified as ‘Undefined’ comprised almost 1/3 of the total number of cells and represented low quality cells (Supp. Fig. 1b).

A specific gene expression profile was also used to determine a phase of the cell cycle of each cell (CellCycleScoring Seurat function) to ensure that the cells in all phases are equally distributed among clusters. Individual cell types were filtered based on the number of genes detected (nFeature_RNA), number of counts (nCount_RNA) and amount of mitochondrial RNA (percent.mt). The cut-offs specific for each cell type of interest were the following: astrocytes—nFeature_RNA > 1000, 2000 < nCount_RNA < 10,000, percent.mt < 8; microglia—nFeature_RNA > 700, 1000 < nCount_RNA < 10,000, percent.mt < 5; oligodendrocytes—nFeature_RNA > 1300, 2500 < nCount_RNA < 50,000, percent.mt < 5 (Supp. Fig. 1d). Tissue dissociation may induce the expression of IEGs, the first rapid cellular response to stimuli^24,30^. A set of the IEGs (e. g. *Fos* and *Jun* transcription factors) was projected onto the UMAP using AddModuleScore function to investigate the level of induction of these genes by sample preparation (Supp. Fig. 1c). The SoupX R package (version 1.5.2)^31^ was applied to remove the contaminating RNA background. The unfiltered and annotated data were supplied as the input. The contamination fraction was estimated by the automated method and was in the range from 1 to 2 % for individual samples. The count values were subsequently corrected for the contamination.

To view the overall differences between samples by pseudobulk principal component analysis (PCA), the normalized and scaled data set was used to create a pseudobulk data by summing up the gene counts of cells belonging to the same condition, age, and sex.

### Differential expression analysis and Gene Set Enrichment Analysis

Differentially expressed genes (DEGs) in the single-cell data set were identified by t-test in Seurat’s FindMarkers function. Normalized and scaled data in the RNA assay were used in this analysis. *P*-adjusted value (*p*_adj_) threshold was set to 0.05, and genes with log_2_ fold change (log_2_FC) > 1 or < − 1 were considered differentially expressed. Males and females were compared at each time point for each cell type and condition. Control (CTRL) and SOD1 pairs were also tested at each time point and for each cell type (end-stage DEGs in Supp. Tab. 1). The Gene Set Enrichment Analysis (GSEA)^32^ was performed using clusterProfiler R package (version 4.0.5)^33,34^. Reference gene set size was limited to 10–800 genes and the significance threshold was set to *p*_adj_ = 0.05. Only results where more than one gene contributed to the enrichment (core enrichment) were considered relevant.

### The analysis of cellular subtypes

Individual cell types of interest (astrocytes, microglia, oligodendrocytes) were analyzed separately. Mitochondrial and ribosomal genes were not included in the subsequent analyses. The filtered, normalized, scaled and SCTransformed data were then subjected to PCA. Within the scope of quality control, several genes were excluded, as they introduced additional undesirable variability in the clustering (*Sod1*, *Gm8566*, *Cmss1*, *Cdk8* and lncRNAs *Xist*, *Gm42418*, *Gm424181*, *Malat1*). 16 PC for microglia and astrocytes, and 17 PC for oligodendrocytes were visualized using UMAP, and clustered at a resolution of 0.2 (FindNeighbors and FindClusters functions). A cluster of male control cells at the 3 M time point was excluded from the astrocyte and oligodendrocyte subsets, as it expressed potentially stress related marker genes (e.g., *Cdkn1a, Fkbp5*), which might have been induced during sample preparation (Supp. Fig. 2).

Markers of the subclusters were identified using the default Wilcoxon test in the FindAllMarkers function (at least 10 % cells in a cluster expressing the given marker, log_2_FC > 0.25, Supp. Tab. 3). The identity of the subclusters was determined by comparison with available gene signatures of various previously described cellular subtypes using the AddModuleScore function and by manual annotation based on the calculated marker genes. Reference gene expression signatures were taken from Habib et al.^5^ (Gfap-Low and Gfap-High astrocytes), Sala Frigerio et al.^4^ (ARM, IRM) and Marques et al.^35^ (MFOL1/2, MOL2, MOL5/6). The intermediate state of astrocytes was visualized using a gene set containing calculated markers of cluster 2 and markers of transition state published by Habib et al.^5^. The signature of homeostatic microglia resulted from the combination of homeostatic markers mentioned in Keren-Shaul et al.^3^, Mathys et al.^36^ and Butovsky and Weiner^37^. The top 30 genes were used for the projection in astrocytes, and 20 genes were used in the microglia and oligodendrocytes. Numbers of cells entering differential expression analysis (DEA) and subpopulation analysis are provided in Supp. Tab. 4.

## Results

### Behavioral tests confirmed ALS-like phenotype

Firstly, we investigated the expected disease-related phenotypic changes characteristic for the animal model used in this study. Our goal was to assess the main turning points of the disease and characterize its progression.

**Figure 1:**
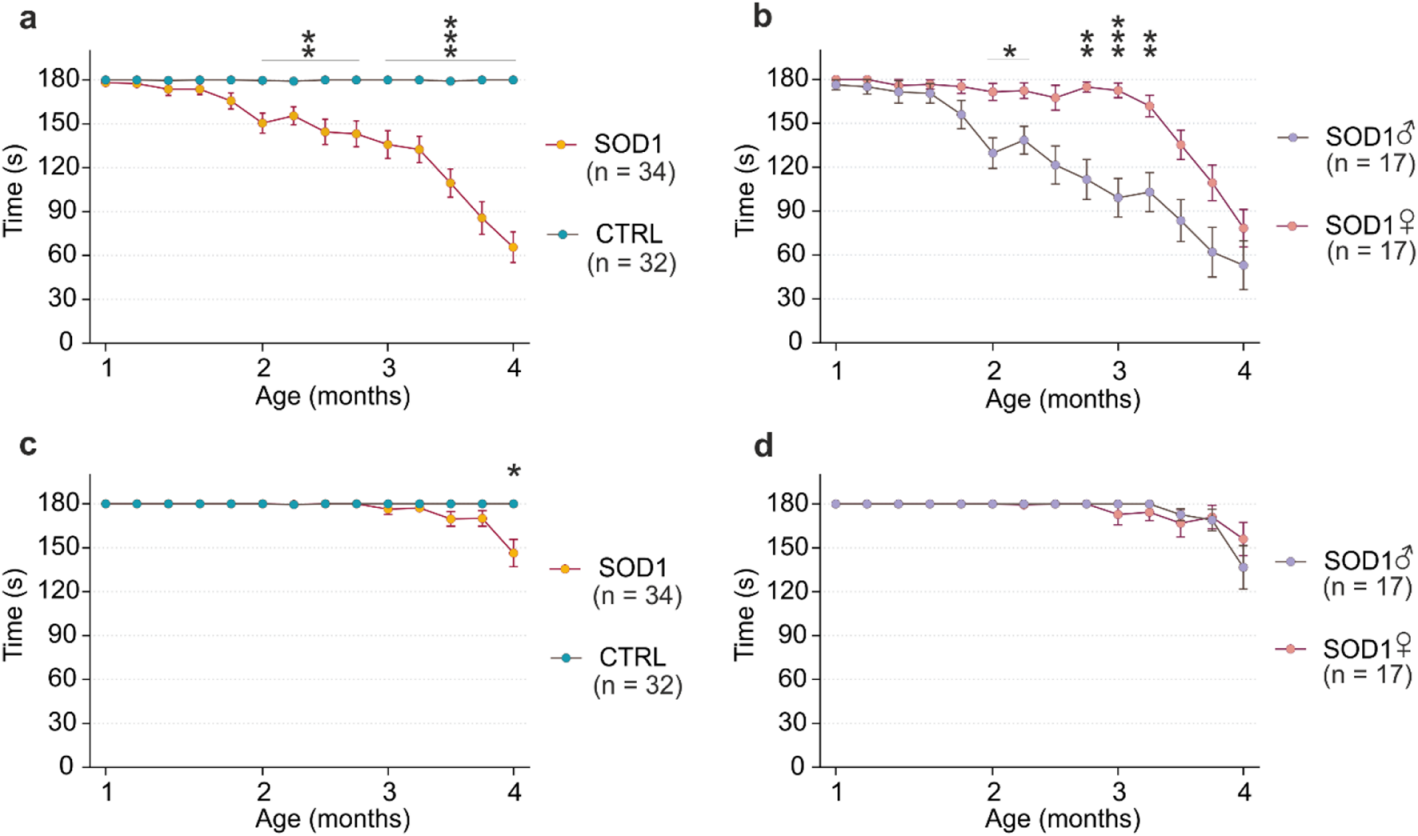
The cortical pathology evaluation by behavioral testing and immunohistochemistry. (**a**) The hanging wire test confirmed the motor skills decline in SOD1 animals during progression compared to the CTRLs. (**b**) Results from the hanging wire test comparing SOD1 animals divided based on sex revealed phenotypical differences in onset. (**c**), (**d**) The rotarod results comparing the CTRL and SOD1 animals did not reveal significant changes in progression. Statistical significance was determined using two-way ANOVA with Holm-Sidak’s post-hoc test, error bars representing SEM. *p_adj_ ≤ 0.05, **p_adj_ ≤ 0.01, ***p_adj_ ≤ 0.001. n states the number of performing animals.

To study the phenotype, we used two types of behavioral testing—the wire grid hang test and the rotarod performance test. We tested comparable groups of animals harboring SOD1(G93A) mutation (SOD1), and control animals (CTRL) with even numbers of males and females within each group. At the beginning of the testing period (1 month), all the animals performed for the maximum time (180 s) in both tests (see “Methods” section). The differences between CTRL and SOD1 animals were first noticeable in the wire grid hang test, where primarily the muscle strength is tested (Fig. 1a). The differences became significant at two months of age, so we considered this point an onset. The performance then slowly declined until a sudden drop at three months, signaling the beginning of the symptomatic stage. This stage, accompanied by an even further performance decline, lasted approximately a month, after which the animals reached the end-stage marked by seriously impaired motor functions. The decrease in motor coordination of the animals measured by the rotarod (Fig. 1c) was only significant at the end-stage.

Investigating the SOD1 male versus female performance, the wire grid hang test revealed differences in the symptom onset and the progression in general (Fig. 1b). The onset in males appeared earlier, and overall they performed worse than the females, which is in agreement with human pathology^38^. However, despite the later onset, the female performance in the symptomatic stage declined faster than the male, resulting in similar results for both sexes at the end-stage. The rotarod measurements did not reveal any significant sex-related differences in motor coordination during the progression.

Thus, behavioral tests confirmed the characteristic features of the model, identified sex-related differences, and determined the four main time points of the disease, which we considered in the following experiments.

### Identical cell populations were identified in the control and SOD1 mouse cortex using scRNA-seq

**Figure 2:**
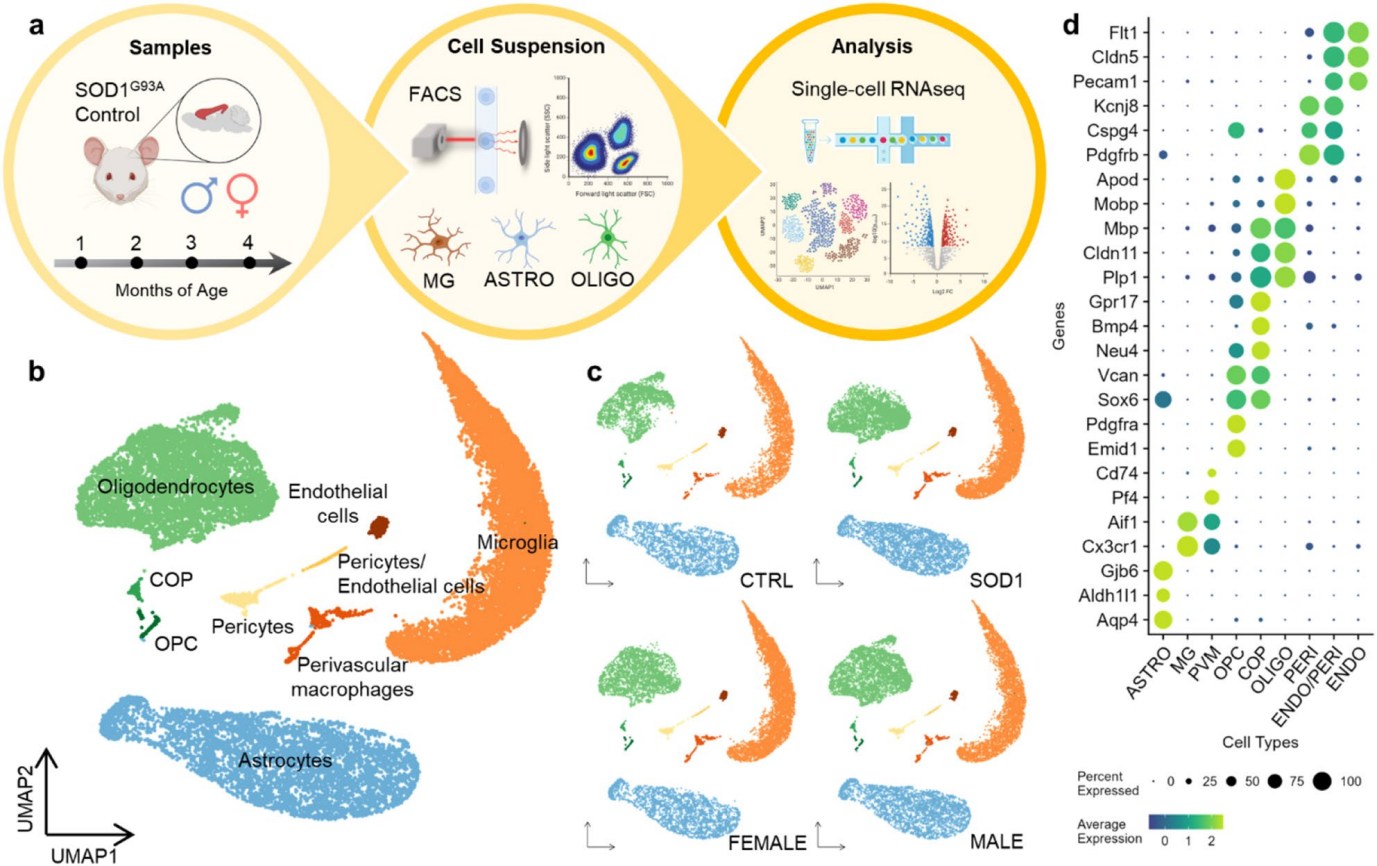
Single-cell RNA sequencing experiment overview. (**a**) A scheme summarizing the process of the sequencing experiment. (Created with BioRender.com) (**b**) A UMAP plot visualization of the identified cell clusters, containing cells from all samples. ASTRO n = 5536, MG n = 8429, OLIGO n = 6180, PVM n = 434, OPC n = 165, COP n = 98, PERI n = 395, ENDO n = 235, ENDO/PERI n = 108, total n = 21,580. (**c**) A visualization of cluster representation and the prevalence of targeted glia in both conditions and sexes. CTRL n = 7646, SOD1 n = 12,499, female n = 10,246, male n = 9899. (**d**) A list of canonical marker genes used for identification of cell clusters. ASTRO—astrocytes, MG—microglia, PVM—perivascular macrophages, OPC—oligodendrocyte precursor cells, COP—committed oligodendrocyte precursors, OLIGO—oligodendrocytes, PERI—pericytes, ENDO—endothelial cells.

The scRNA-seq experiment was designed as follows (Fig. 2a): CTRL and SOD1 mice were sacrificed at four time points, representing the main stages of the disease, with two males and two females used per condition at each time point. All cell suspensions were prepared from the motor and somatosensory cortical tissue and were enriched for three glial cell types—astrocytes, microglia, and oligodendrocytes—using FACS.

The scRNA-seq data followed an initial quality control, filtering, and clustering, and the resulting set of single cells was annotated using the expression of canonical marker genes of individual glial cell populations (Fig. 2b,d). As expected, the most numerous clusters in the data set were identified as astrocytes (*Aqp4*, *Aldh1l1*, *Gjb6*), microglia (*Cx3cr1*, *Aif1*), and oligodendrocytes (*Mobp*, *Apod*). Oligodendrocyte precursor cells (OPC) and committed oligodendrocyte precursor cells (COP) clustered separately from the mature oligodendrocytes that prevailed in the data set. The marker genes of these populations partially overlapped, indicating a gradual maturation of OPCs into oligodendrocytes (*Emid1*, *Pdgfra*, *Sox6*, *Vcan*, *Plp1*, *Cldn11* and others). In addition, perivascular macrophages, pericytes, and endothelial cells were present in the minority.

Each cell was assigned a sex identity based on the expression of X and Y chromosome-associated genes. Cells that did not fulfil the criteria for sex determination (see *Methods,* Supp. Fig. 1a, b) were excluded from further analyses. No cell cluster was overrepresented specifically in CTRL or SOD1, or in the male or female samples, confirming the robustness of cell preparation (Fig. 2c). Furthermore, the low proportion of mitochondrial reads and minimal activation of immediate early genes further validated the data quality (Supp. Fig. 1c, d). Overall, we successfully identified the targeted cell populations in the dataset and observed their equal proportions in both conditions and sexes.

### The cortical glia of SOD1 mice showed minor changes in gene expression at the late stage of the ALS-like pathology

**Figure 3:**
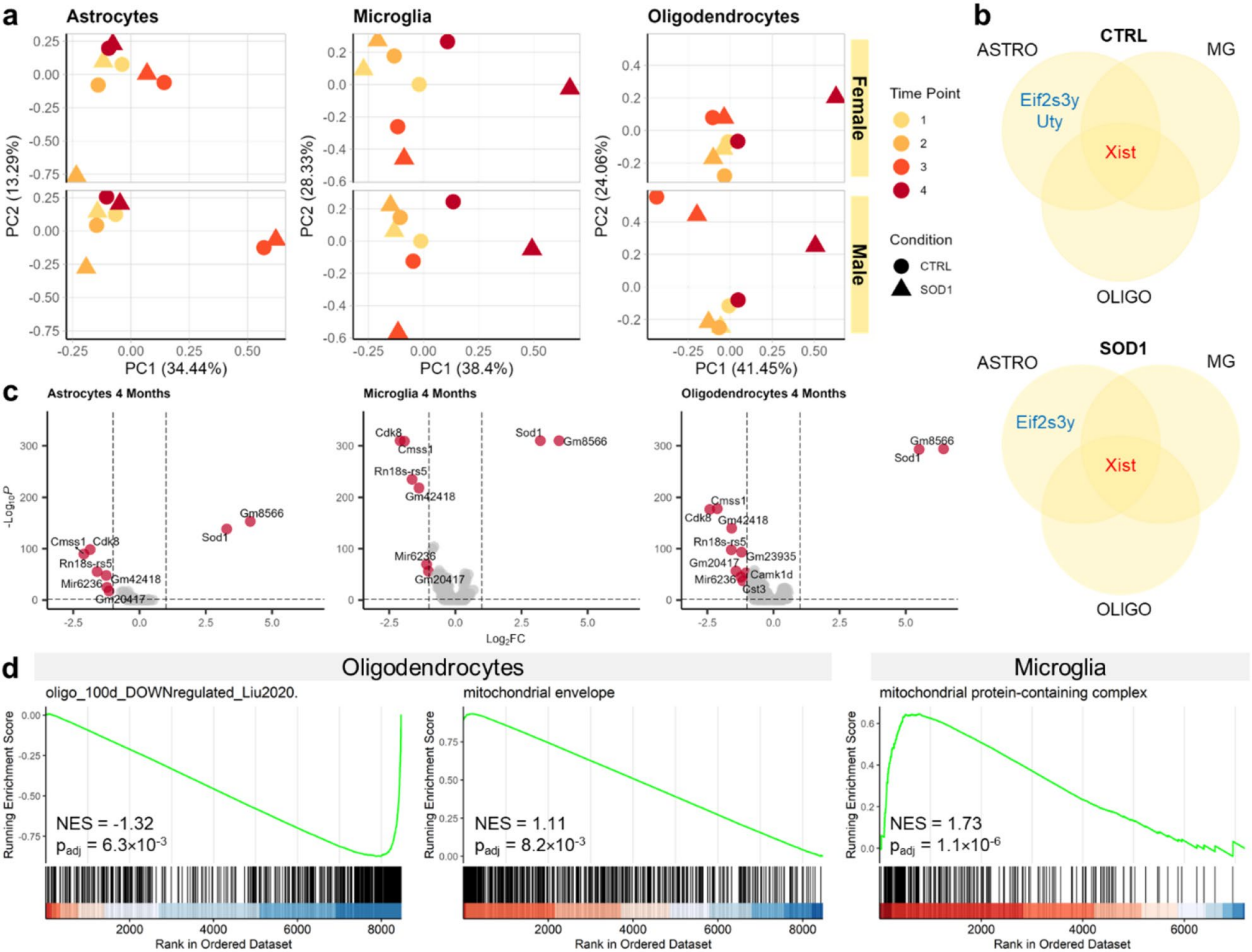
Pseudobulk and differential expression analysis reveals only minor changes in gene expression. (**a**) Pseudobulk PCA clustering comparison of male and female samples showing a shared reaction to the ongoing pathology in microglia and oligodendrocytes at 4 M. (**b**) A visualization of the few differentially expressed genes found dysregulated in males and females in all cell types at 4 M time point. *Xist* clearly distinguishes female cells, whereas *Eif2s3y* and *Uty* are both expressed by male chromosome Y. (**c**) Results of the DEA comparing CTRL and SOD1 4 M samples show a limited number of up- and downregulated genes at the final stage. (**d**) Enrichment curves visualising the results of the GSEA at 4 M. One reference gene set was enriched among genes downregulated in oligodendrocytes. Analysis of GO terms showed mitochondrial components to be enriched among genes upregulated in oligodendrocytes and microglia.

To provide a general view on the gene expression changes in SOD1 mice, each of the main cell types was subjected to PCA as a pseudobulk (Fig. 3a). The analysis revealed a minor alteration between the CTRL and SOD1 samples in both sexes during disease progression. The first apparent shift between the samples appeared at four months of age in microglia and oligodendrocytes, suggesting their reaction to ALS-like pathology. The SOD1 astrocytes, frequently reported to be activated in SOD1(G93A) and other models of ALS^14,18,19,39^, remained unchanged and clustered with the CTRL samples. Notably, the clustering showed a displacement of three-month-old (3 M) male data points. This was most prominent in astrocytes, where it represented the highest source of variability (reflected by separation in PCA1). Exploring the source of the variability, we identified a minor cluster of cells within astrocytes and oligodendrocytes, which was only present in 3 M CTRL males (Supp. Fig. 2). The cluster was characterized by the expression of stress-related genes *Cdkn1a* and *Fkbp5*, which had the most extreme values of loadings in respective principal components (PCs) in the pseudobulk analysis, confirming the effect of the cluster on the displacement of 3 M CTRL male data points. As the presence of the cluster had a negligible effect on further analysis, we considered it as a technical artifact of sample processing and removed it from the dataset. This finding, however, showed the power of the single-cell analysis to characterize even minor changes in cell subpopulations, which might otherwise be hard to interpret in traditional bulk analysis.

Based on the sex-related differences in behavioral tests (Fig. 1a–d), we examined the potential ALS-associated gene expression variations between the sexes by DEA, and compared the male and female cells for CTRL and SOD1 separately. The analysis yielded only a few DEGs based on the set thresholds of |log_2_FC|> 1 and *p*_adj_ < 0.05. The X chromosome gene *Xist* was significantly upregulated in the females in all three cell types, regardless of genotype. The *Xist* gene plays a major role in the gene dosage compensation in females by silencing one of the X chromosomes, and is therefore expressed only in female cells^40^. Two genes encoded by chromosome Y (*Eif2s3y*, *Uty*) were upregulated in males, but the difference in their expression only exceeded the log_2_FC threshold in astrocytes (Fig. 3b). Apart from these, no other dysregulated genes related to the pathology progression and sex were found in our data.

To investigate disease-related transcriptional changes, we performed the DEA on the comparisons of CTRL and SOD1 samples at each time point and for each cell type, considering males and females together. The *Sod1* gene was the only one significantly upregulated DEG in all the measured stages of the disease, including the end-stage as shown in Fig. 3c and Supp. Tab. 1 (|log_2_FC|> 1, *p*_adj_ < 0.05), confirming the validity of the SOD1(G93A) model. Other dysregulated genes were mostly noncoding or ribosomal transcripts that evaded quality control. Additionally, the 4 M CTRL samples were marked by an increased expression of genes *Cdk8* and *Cmss1* across all cell types. These two genes were identified in the subsequent analysis as confounders negatively effecting sub-clustering results (Supp. Fig. 2a, b). Therefore, they were considered as biasing factors without connection to the ALS-like pathology. Together, these results show minimal variation in gene expression related to the pathology progression in the cortex of the SOD1(G93A) mouse, regardless of sex, with the indication of subtle changes in microglia and oligodendrocytes in the late phase of the disease.

### The GSEA indicated an altered mitochondrial function in cortical microglia and oligodendrocytes of SOD1 mice

To investigate the potential biological significance of the minimal changes in gene expression detected by DEA, we utilized the GSEA^32^ focused on the most affected 4 M time point. As GSEA considers the expression of all genes, regardless of cut-offs in log_2_FC or p-value, it allows for the finding of any dysregulated processes even if the change of the individual genes is minor.

First, we employed a meta-analysis approach, and collected the gene signatures of glial cells affected by SOD1 mutation from 15 transcriptomic studies published in the last 20 years (Supp. Tab. 2). This set includes data mostly derived from the spinal cord of the SOD1(G93A) model. Using GSEA, we detected the enrichment of only a single gene set that was recently reported by Liu, et al.^15^ as downregulated in brainstem oligodendrocytes in 100-day-old mice. In concordance, our results showed a negative enrichment in oligodendrocytes (NES = − 1.32, *p*_adj_ = 6.3 × 10^− 3^; NES—normalized enrichment score; Fig. 3d), indicating small, but significant changes in the cortical 4 M SOD1 oligodendrocytes. No gene set was found enriched for microglia or astrocytes.

In addition to the meta-analysis, we also looked for Gene Ontology (GO) terms ^41,42^ enriched in our data. Two activated terms related to mitochondria turned out to be upregulated in our data set: *mitochondrial envelope* in oligodendrocytes (NES = 1.11, *p*_adj_ = 8.2 × 10^–3^) and *mitochondrial protein-containing complex* in microglia (NES = 1.73, *p*_adj_ = 1.1 × 10^–6^) (Fig. 3d). In support of our data, mitochondrial dysfunction has been extensively discussed as one of the factors contributing to ALS pathology, not only in relation to the mutant *Sod1* gene, but also to other ALS-linked genetic perturbations (reviewed in Jankovic et al.^43^).

Taken together, despite the low number of DEGs, we identified subtle changes in the mitochondria function in cortical oligodendrocytes and microglia. However, considering the number of enriched terms and their significance, the severity of the dysfunction is rather low.

### The subpopulation analysis confirmed subtle changes in oligodendrocytes and microglia

As the pseudobulk analyses revealed only subtle variations in the gene expression of the SOD1(G93A) cortical glia, we speculated that the pathological changes could be represented by a small fraction of cells, hidden at the population level. Therefore, we conducted an in-depth sub-clustering analysis with the goal to identify subpopulations potentially playing a role in the disease progression.

**Figure 4:**
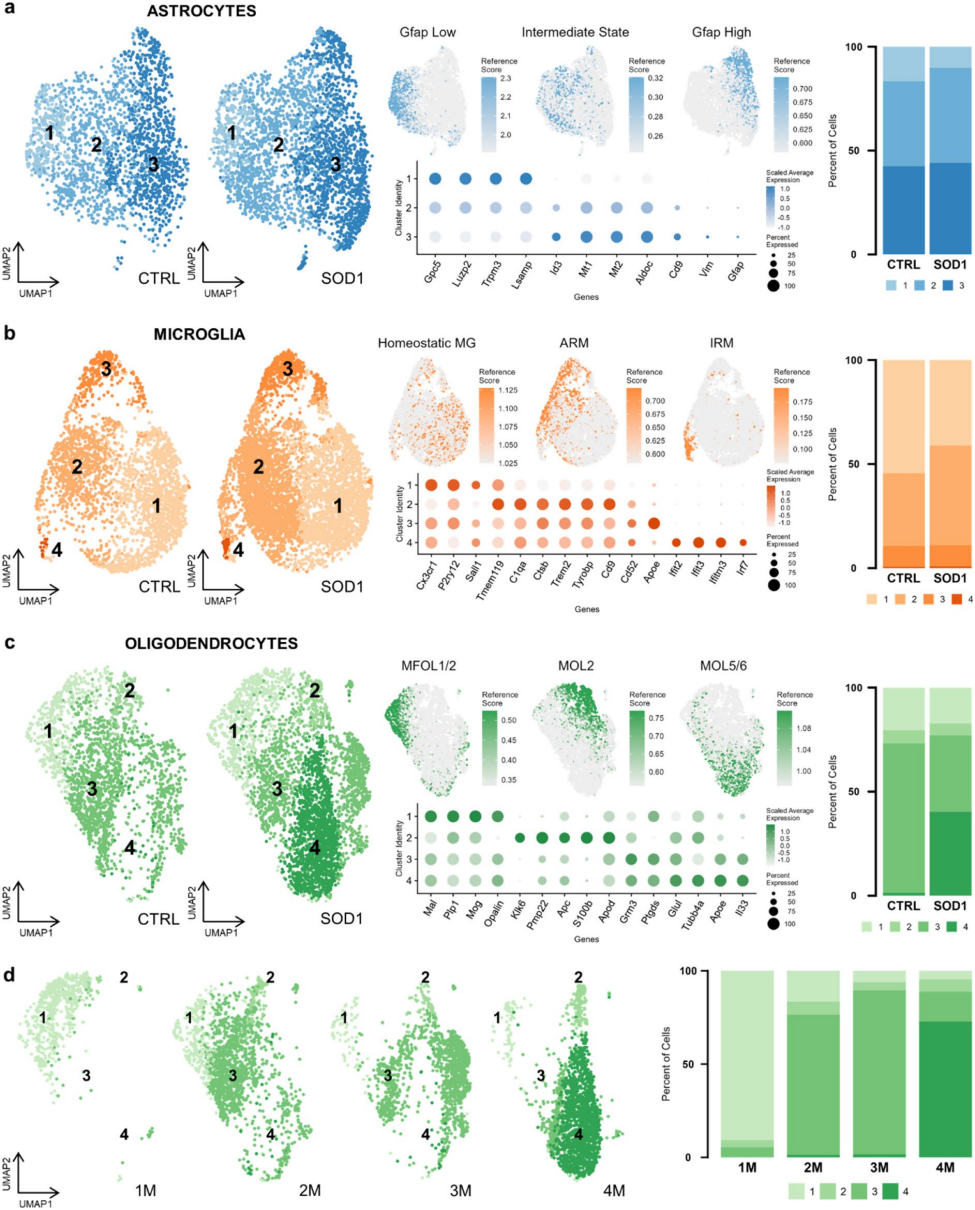
The subpopulation analysis revealed a unique subpopulation of oligodendrocytes in SOD1 samples. UMAP visualization of subpopulations of astrocytes (n = 5292) (**a**), microglia (n = 8414) (**b**), and oligodendrocytes (n = 5876) (**c**) split to CTRL and SOD1 condition (left), including cells from all four time points. Gene expression signatures of previously described subpopulations are shown projected onto UMAP (middle-top). A list of representative cluster markers used for their annotation (middle-bottom). Proportions of subpopulations in CTRL and SOD1 (right), including cells from all four time points. (**d**) UMAP visualization of CTRL and SOD1 oligodendrocytes split according to age (1 M: n = 682, 2 M: n = 1697, 3 M: n = 1430, 4 M: n = 2067). Bar plot shows proportions of subpopulations at each time point.

Starting with astrocytes, we identified three clusters that were present in both the CTRL and SOD1 samples in a similar proportion (Fig. 4a). To annotate these clusters, we calculated their marker genes (Supp. Tab. 3) and visualized the gene signatures of astrocytic subtypes described by Habib et al.^5^ in a mouse model of Alzheimer’s disease (AD). The study identified two similar subpopulations of astrocytes expressing markers of reactivity –⁠ Gfap-High and DAAs. While Gfap-high were present in controls and in AD samples, DAAs were unique to the AD model, and the authors suggested their potential role in the disease progression. Marker genes of cluster 3 in our data partially overlapped with the markers of the Gfap-High cluster (e. g. genes *Mt1*, *Mt2*, *Id3*, *Cd9*, *Vim*), but we did not detect the DAAs specifically in SOD1 samples. Of note, *Gfap* and *Vim* expression levels were low, suggesting a limited pathological reaction of astrocytes in the cortex of the mutant SOD1(G93A) mice. This finding complies with the results of the pseudobulk analysis, showing no changes in astrocytes. Cluster 1 shared common genes with Gfap-low astrocytes (e. g. *Luzp2*, *Trpm3*), and the remaining cluster 2 expressed markers of both Gfap-High and Gfap-Low clusters, therefore representing an intermediate state cluster.

Microglia were grouped into four clusters as shown in the UMAP plot in Fig. 4b. Cluster 1 was characterized by the expression of homeostatic markers^3,36,37^. The gene signatures of microglia in clusters 2 and 3 resembled the expression profile of ARM, that were described in a model of AD, but also in a smaller proportion in a healthy brain^3,4^. These activated microglia characteristically downregulate homeostatic genes such as *Cx3cr1*, *P2ry12,* and *Sall1*, which is also noticeable in our data (Fig. 4b). Additionally, cluster 3 was marked by a higher expression of *Apoe*, which is a major regulator of microglial neurodegenerative phenotype^44^. Cluster 4 represented IRM^4^, and was clearly distinguishable by the expression of *Ifit2*, *Ifit3,* and *Ifitm3*, the genes involved in the interferon response pathway. However, the proportion of IRM was very low in both conditions. Nevertheless, we detected a small increase in the subpopulation of activated microglia in SOD1 samples (cluster 2), suggesting a starting activation of microglia in response to pathological stimuli.

Oligodendrocytes formed four clusters (Fig. 4c). Cluster 1 shared a similar gene expression signature with myelin forming oligodendrocytes (MFOL) described by Marques, et al.^35^, including the expression of *Mal*, *Plp1*, *Mog,* and *Opalin* genes. These cells were predominantly present at the one month (1 M) time point (Fig. 4d), which coincides with the extensive myelination in rodents during the early weeks after birth^45^. Cluster 2 represents mature oligodendrocytes (MOL2) expressing *Klk6*, but also mature oligodendrocyte marker *Apod* and genes typical for myelinating cells *Pmp22*, *S100b,* and *Apc*^7,35,46^. Floriddia et al.^7^ reported the MOL2 population to be more abundant in the white matter of the spinal cord, but also to a lesser extent in the cortex^35^. The proportions in both conditions were similar for clusters 1 and 2. As for clusters 3 and 4, they showed an increased expression of several genes found in mature oligodendrocyte populations MOL5/6^7,35^. Interestingly, cluster 4 was present almost exclusively in the SOD1 samples (Fig. 4d), and it was characterized by a higher expression of *Apoe* and *Il33*. *Il33* is upregulated in oligodendrocytes and astrocytes in lesions and can be released by stressed or damaged cells. It has an anti-inflammatory effect and promotes the activation of microglia (reviewed in Sun et al.^47^). Upregulation of *Ill33* has been previously reported in disease-associated oligodendrocytes in a mouse model of AD^48,49^. *Apoe* was identified as a marker gene of immune oligodendroglia (ImOLG)^50^, which were enriched in multiple sclerosis lesions, suggesting their role in chronic demyelination and continuous attempts to remyelinate^51^. Thus, we hypothesize that cluster 4 represents a damaged or reactive state of oligodendrocytes, responding to the pathological stimuli in the 4 M SOD1 cortex. It is tempting to speculate whether the small increase in numbers of activated microglia and possibly the shift to the intermediate state in astrocytes that we observed in the SOD1 (Fig. 4a,b, respectively) might be a response to the damaged/activated oligodendrocytes. This kind of interaction between disease-associated states of glial cells was recently described in AD^52^, and also in ALS^2^.

Overall, we were able to identify multiple subpopulations of astrocytes, microglia, and oligodendrocytes, and we recognized the gene expression patterns of specific, previously described, cellular subtypes. However, in contrast with our expectations, we did not detect any disease-associated subpopulations in the SOD1 samples, apart from the damaged/activated oligodendrocytes.

### The immunohistochemical identification of changes in the cortical and spinal glia of SOD1 mice

**Figure 5:**
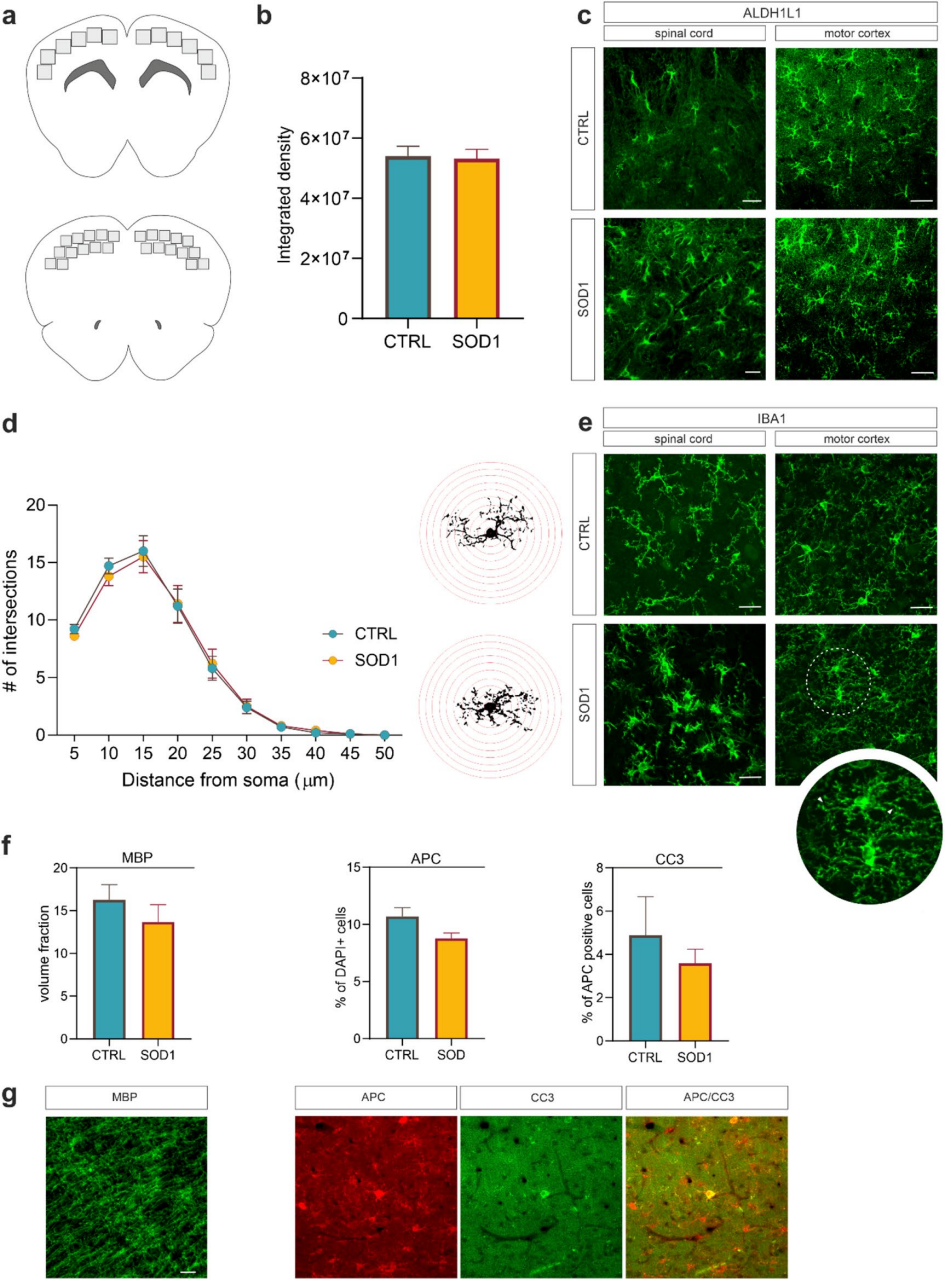
The immunohistochemistry (**a**) An upper cartoon depicts 12 areas scanned for the investigation of morphological changes and quantification of APC and CC3. Lower cartoon shows 24 areas scanned for the MBP analysis. (**b**) Fluorescence analysis did not reveal any significant differences in morphology between cortical astrocytes in SOD1 (n = 6) and CTRL (n = 6) animals. (**c**) Representative pictures of ALDH1L1 staining in cortex and spinal cord comparing astrocytes show morphological difference between SOD1 and CTRL in the spinal cord but no noticeable difference in the cortex. (**d**) The results of Sholl analysis indicated very similar microglia complexity in both CTRL (n = 6) and SOD1 (n = 6) samples. Scholl masks with red concentric radii depict single thresholded microglia as they were used for the analysis. (**e**) Representative images of cortical and spinal slices stained with IBA1 show evident amoebic morphology of spinal microglia but only subtle morphological changes represented by *bulbous termini* (see close up) in the cortex. (**f**) The quantification of MBP in the cortex revealed insignificant difference between SOD1 (n = 6) and CTRLs (n = 6). The number of APC + cells was consistent between SOD1 (n = 3) and CTRLs (n = 3) and the number of APC + cells co-stained with CC3 used as a marker of apoptosis in oligodendrocytes also remained similar, suggesting no apparent oligodendrocyte degenerations. (**g**) Representative images of MBP, APC, and CC3 staining Scale bars, 20 μm. The statistical significance was determined using unpaired t-test. Error bars represent SEM. n states the number of used animals.

The results of the scRNA-seq analysis suggested minor changes in the cortical glia of the SOD1(G93A) mouse model. To further explore and validate this conclusion, we conducted an immunohistochemical analysis of glia in the motor and primary somatosensory cortex (the identical region used for the sequencing) at the 4 M time point, when the phenotypic changes are the most pronounced. The imaged zones within areas of interest are represented in Fig. 5a. As the morphological changes are well described in the spinal cord, we also stained the lumbar region of the spinal cord at the end-stage (4 M), and used those pictures as a reference for advanced gliosis in our animals.

Astrocytes were stained using ALDH1L1 marker, which allowed the inspection of the whole cell morphology, including the processes. Astrogliosis, a morphological change marked by enlargement of the cellular body and shortened, thickened processes, is a characteristic response of astrocytes in pathological states, and it was well described in the ALS spinal cord^13,14^. In the cortex we conducted fluorescence analysis in order to find any morphological differences (Fig. 5b), but the analysis showed very similar fluorescence values of both SOD1 and CTRL astrocytes, suggesting there is no cell enlargement associated with activation. The similar morphology can be seen in Fig. 5c in comparison with astrocytes in the ventral horns of the spinal lumbar region, which showed an enlarged body with distinctively shortened processes. The results from fluorescence analysis align with little or no change detected in astrocytes at the level of gene expression.

Microglia, like astrocytes, change their morphology in response to pathological stimuli. They retract processes and adopt a specific amoebic shape, typical for their activated state. To visualize microglia, we used IBA1 antibody and stained both the cortex and lumbar spinal cord for comparison. The Sholl analysis we used to evaluate the changes in morphology (Fig. 5d) is frequently conducted to quantify the complexity of cell´s arborization. The analysis confirmed that the SOD1 cortical microglia morphology does not significantly differs from CTRL animals. We did not detect shorter processes or reduced branching, which would suggest activated phenotype. However, during the analysis we noticed some tips of the processes looking bulbous and enlarged (Fig. 5e). These structures, called *bulbous termini*, appear as the microglia’s first reaction after injury^53^, and may represent an initial phase of microglia activation observed in the transcriptomic data. The SOD1 microglia in the ventral horns of the spinal cord on the other hand display the typical amoebic shape with very short and thickened processes associated with activation.

The previous analysis identified a SOD1-specific oligodendrocyte cluster (cluster 4) suggesting a certain portion of cells as being apoptotic, we thus focused on the protein expression changes related to chronic demyelination, necrosis, or apoptosis. Accordingly, we stained for myelin basic protein (MBP)—a marker of myelination, adenomatous polyposis coli (APC)—a marker of adult oligodendrocytes, and cleaved caspase 3 (CC3)—an apoptotic marker. The quantification of MBP signal intensity as well as the number of APC + oligodendrocytes in the cortex of the SOD1 animals excluded the demyelination processes as there was no significant decrease of MBP or APC + cells compared to the CTRLs (Fig. 5f). Similarly, the co-staining of CC3 with APC did not reveal a different abundance of CC3 + oligodendrocytes in either the SOD1 or CTRLs, suggesting a similar rate of apoptosis (Fig. 5f). MBP staining and representative image of a cell positive for APC and CC3 can be found in Fig. 5g. Collectively, the data showed no significant demyelination or degeneration of oligodendrocytes, therefore it is likely that the SOD1-specific cluster 4 identified in scRNA-seq in 4 M animals does not represent dying or damaged cells.

Taken together, we did not detect any profound morphological changes in the cortical glia of the SOD1 mice. Oligodendrocytes did not show an increased cell death or loss of MBP protein, suggesting its maintained function in the cortex of the ALS mice.

## Discussion

ALS is a devastating neurodegenerative disease with fast progression and no effective treatment strategies. Although it is primarily recognized as a motor-neuron disease, the other cell types, including glial cells, contribute to the disease progression and thus represent a potential target for future therapies. In the past, multiple experimental models have been developed to understand the mechanisms of the disease, as well as to facilitate the search for therapeutic targets. Among them, the SOD1(G93A) mouse model has the prime position as the best-characterized mouse strain used in current ALS research. Despite its long-term application, the effect of the pathological changes across different CNS regions is still not fully understood.

The pathological effect on cortex is of special interest due to its role in the planning, control, and execution of voluntary movements, which are severely affected by ALS. Pathological changes in the cortex of ALS patients have been reported since the first description of the disease in 1869, and the monitoring of cortical structural abnormalities became a standard ALS diagnostic procedure^54^. Along with cortical MN death, changes in glia have also been observed, including microgliosis^11,55–57^, accompanied by the DAM-like gene expression signature^56^, demyelination^8^, and change of oligodendrocyte function from myelinating to neuro-supportive^57^.

While the changes are well documented in humans, less is known about the effect of the ALS-like pathology on the cortex of the SOD1 models. In the SOD1(G93A) model, the cortical MN degeneration was observed early by Özdinler et al.^17^ and others, together with the changes in glia^18–20^. Reactive astrocytes in the SOD1(G93A) cortex were shown to differ from their spinal counterparts, while still maintaining a toxic effect on neurons^18,19,58^. However, Gomes et al.^19^ also reported no significant gene expression changes in microglia or oligodendrocytes. Moreover, others suggested the pathology in the SOD1 model as being restricted to only spinal and bulbar motor neurons, not affecting the motor cortex^21^. Thus, such opposing data raise questions as to what extent the cortex is affected in the SOD1 mouse model, and how well this model recapitulates the cortical pathology in humans.

To fill this knowledge gap, we applied single-cell RNA sequencing supported by immunohistochemistry to detect gene expression changes, the presence of disease-associated cell populations, and morphological or other pathological modifications in cortical glia accompanying the ALS-like pathology in the SOD1(G93A) mouse model. The ALS-like phenotype was confirmed by behavioral testing, including sex-dependent differences in disease progression as reported by McCombe and Henderson^38^. The results of scRNA-seq focused on glia revealed minor changes in microglia and oligodendrocytes, and showed no significant ALS-related shift in astrocytes, which contradicts several studies reporting the reactive phenotype of astrocytes^18,19,58^. Although the only significantly dysregulated gene in the SOD1(G93A) model was *Sod1*, GSEA indicated the mitochondrial dysfunction in microglia and oligodendrocytes. Similar changes have recently been observed by Liu et al.^15^ in the oligodendrocytes isolated from the brainstem of 100-day-old SOD1 mice, suggesting the possible dysregulation of energetic pathways.

As our data were generated within the motor and primary somatosensory cortex, representing an end-point region of corticospinal tract affected by ALS, they allow basic questions to be addressed regarding the site of origin and the direction of ALS progression. Currently, there are two hypotheses, both supported by lines of evidence (reviewed in van den Bos et al.^59^). The ‘dying forward’ hypothesis suggests ALS originates in the cortex and spreads towards the spinal MNs. On the other hand, the ‘dying back’ hypothesis proposes ALS begins within muscles or neuromuscular junctions. Considering the advanced ALS-like phenotype of the end-stage SOD1 mice and the more pronounced changes in the brainstem and the spinal cord observed elsewhere, the milder changes in the cortex indicated by our data are more in favor of the ‘dying back’ hypothesis in SOD1(G93A) mice. Interestingly, this contrasts with a report from Burg et al.^60^, which indicates the cortex as the initiating point of ALS in SOD1(G86R) mice. The contradictory data might suggest a differential effect of specific point mutations on the character of the disease, requiring further investigation.

Many of the previous reports exploring the effect of the SOD1 mutation on motor cortex relied on measuring the limited number of genes and proteins^19,20,55^, or analysis of the bulk population of cells^18,61^. This consequently decreased the sensitivity of the analysis and limited the interpretability of the data. In this study, we used high-throughput scRNA-seq, allowing for an in-depth analysis of small cell populations that cannot be distinguished by bulk approaches. Using scRNA-seq, recent seminal studies revealed various disease-associated populations of glia, playing a major role in the disease progression^3–6^. Interestingly, many of these populations are present in multiple diseases, suggesting common mechanisms employed by glial cells in response to pathological stimuli. For example, a DAM-like population was identified not only in Alzheimer’s disease, but also in the spinal cord of the SOD1(G93A) mouse^3^, and in a spinal cord injury model^62^. In our work, we searched for these populations without any success, which confirmed the minimal changes observed at bulk transcriptional level, as well as by immunohistochemistry. We mostly found similar cellular composition between the control and SOD1(G93A) animals, except for microglia and oligodendrocytes. In the case of microglia, we observed a little increase in the proportion of the activated microglia, suggesting a starting phase of their activation. The data were completed by immunohistochemistry revealing *bulbous termini* on microglial processes. More apparent changes were observed in oligodendrocytes, where sub-clustering analysis identified the existence of a unique population of oligodendrocytes, characterized by an increased expression of *Apoe* and *Il33*, and enriched specifically in 4 M SOD1 samples. Considering no profound loss of oligodendrocytes or a higher apoptotic rate in immunohistochemistry, we could speculate about the active role of this population of oligodendrocytes in disease progression. This is also in line with recent evidence suggesting the active role of oligodendrocytes in the progression of multiple sclerosis^6^ and in aging white matter^63^, which contrasts with their widely accepted passive role. Notably, we did not observe any expression patterns similar to previously reported disease-associated oligodendrocytes^6,48–50^, except *Apoe* or *Il33* mentioned above. However, this might be related to the overall mild changes in gene expression observed throughout our data.

In conclusion, our study demonstrates that cortical glia are only subtly affected in the SOD1(G93A) model, even at the very end-stage of disease. Furthermore, owing to the power of scRNA-seq, we showed that oligodendrocytes potentially actively participate in the pathology, emphasizing the importance of addressing their role in further research. Finally, on reflection of our results, we suggest that the SOD1(G93A) mouse model does not fully recapitulate the human disease and we recommend using a different model system for studying the cortical ALS pathology.

## Conclusions

Collectively, our results suggest the ALS-like pathological changes present in the sensorimotor cortex of SOD1(G93A) mice are minimal. There is an ongoing discussion in the field whether the model mimics the disease completely including the pathology occurring in the cortex and the published results are disputable. Our collective findings inspecting glial cells on multiple levels did not reveal any significant changes of SOD1 astrocytes and only subtle changes of microglia and oligodendrocytes at the final stage. The changes of microglia and oligodendrocytes suggest starting activation linked to the pathology, but the extent of change does not correspond to the pathology described in human tissue. The SOD1 specific oligodendrocytes identified at the final stage, however, contribute to the recently published evidence reporting their active role in neurodegeneration, largely rejecting long-lasting dogma presenting oligodendrocytes as solely passive in the CNS diseases. However, despite the signs of activation, the effect of ALS-like pathology on the glial cells in the cortex of SOD1(G93A) mice is minor and our data provide supporting evidence against the use of this model for studying cortical pathology of ALS.

## Supplementary Information

Supplementary Information 1.

Supplementary Information 2.

## Data Availability

The scRNA-seq data generated during this current study are available in NCBI’s Gene Expression Omnibus^64^ and are accessible through GEO Series accession number GSE206330 (https://www.ncbi.nlm.nih.gov/geo/query/acc.cgi?acc=GSE206330).
