# Supplementary material for: Cortical glia in SOD1(G93A) mice are subtly affected by ALS-like pathology
Source: Sci Rep. 2023 Apr 21;13:6538. doi: 10.1038/s41598-023-33608-y (PMC10121704; doi:10.1038/s41598-023-33608-y)
Supplement: Supplementary file 1 — Supplementary Information 1. [file 41598_2023_33608_MOESM1_ESM.docx]

**Supplementary Material**

|  | **Gene** | **p_val** | **avg_log2FC** | **pct.1** | **pct.2** | **p_val_adj** |
| --- | --- | --- | --- | --- | --- | --- |
| **ASTROCYTES** | Cmss1 | 5.14E-95 | -2.10 | 0.88 | 0.99 | 2.39E-90 |
|  | Cdk8 | 8.55E-104 | -1.86 | 0.80 | 0.99 | 3.98E-99 |
|  | Rn18s-rs5 | 7.69E-61 | -1.60 | 0.73 | 0.94 | 3.58E-56 |
|  | Gm42418 | 2.81E-53 | -1.26 | 1.00 | 1.00 | 1.31E-48 |
|  | Mir6236 | 2.70E-30 | -1.23 | 0.56 | 0.87 | 1.26E-25 |
|  | Gm20417 | 5.36E-23 | -1.15 | 0.44 | 0.75 | 2.50E-18 |
|  | Sod1 | 8.37E-144 | 3.29 | 0.92 | 0.43 | 3.90E-139 |
|  | Gm8566 | 1.28E-158 | 4.18 | 0.93 | 0.00 | 5.97E-154 |
| **MICROGLIA** | Cdk8 | 0.00E+00 | -2.09 | 0.75 | 1.00 | 0.00E+00 |
|  | Cmss1 | 0.00E+00 | -1.93 | 0.91 | 1.00 | 0.00E+00 |
|  | Rn18s-rs5 | 6.41E-240 | -1.64 | 0.80 | 0.99 | 2.98E-235 |
|  | Gm42418 | 2.06E-223 | -1.38 | 1.00 | 1.00 | 9.60E-219 |
|  | Mir6236 | 4.94E-75 | -1.09 | 0.61 | 0.89 | 2.30E-70 |
|  | Gm20417 | 1.57E-61 | -1.02 | 0.17 | 0.61 | 7.31E-57 |
|  | Sod1 | 0.00E+00 | 3.21 | 0.98 | 0.27 | 0.00E+00 |
|  | Gm8566 | 0.00E+00 | 3.92 | 0.98 | 0.00 | 0.00E+00 |
| **OLIGODENDROCYTES** | Cdk8 | 5.33E-182 | -2.41 | 0.79 | 1.00 | 2.48E-177 |
|  | Cmss1 | 2.49E-183 | -2.12 | 0.95 | 1.00 | 1.16E-178 |
|  | Rn18s-rs5 | 2.44E-103 | -1.60 | 0.90 | 1.00 | 1.14E-98 |
|  | Gm42418 | 2.06E-145 | -1.58 | 1.00 | 1.00 | 9.57E-141 |
|  | Gm20417 | 6.94E-62 | -1.42 | 0.32 | 0.86 | 3.23E-57 |
|  | Mir6236 | 2.44E-50 | -1.23 | 0.43 | 0.88 | 1.13E-45 |
|  | Gm23935 | 1.14E-98 | -1.21 | 0.87 | 1.00 | 5.31E-94 |
|  | Cst3 | 8.42E-43 | -1.16 | 0.00 | 0.69 | 3.92E-38 |
|  | Camk1d | 2.10E-58 | -1.04 | 0.71 | 0.97 | 9.78E-54 |
|  | Sod1 | 2.57E-298 | 5.51 | 1.00 | 0.57 | 1.20E-293 |
|  | Gm8566 | 0.00E+00 | 6.43 | 1.00 | 0.00 | 0.00E+00 |

**Supplementary Table 1**

**DEA results.** Genes differentially expressed in 4M SOD1 samples in all three cell types, calculated by FindMarkers function using t-test (Bonferroni correction). The significance thresholds were set to |log_2_FC| > 1 and p_adj_ < 0.05.

| **Study** | **Model** | **Sex** | **CNS Region** | **Time Points** | **Cell Types** | **Labelling** | **Collection** | **Analysis** |
| --- | --- | --- | --- | --- | --- | --- | --- | --- |
| Ferraiuolo, et al. ^1^ | SOD1(G93A) | male | lumbar SC | 60 days | ASTRO | ALDH1L1 | LCM | microarray |
| Sun, et al. ^2^ | SOD1(G37R) | - | SC | 8, 10.5 months | ASTRO, OLIGO | EGFP | TRAP (Aldh1l1, Cnp1) | RNA-seq |
| Phatnani, et al. ^3^ | SOD1(G93A) | both | SC (culture) | - | primary ASTRO | - | - | RNA-seq |
| Miller, et al. ^4^ | SOD1(G93A) | - | entire cortex | 120 days | ASTRO | ALDH1L1 | FACS | microarray |
| Liu, et al. ^5^ | SOD1 (G93A) | female | brainstem | 100 days | all | - | - | sc-RNAseq |
| Keren-Shaul, et al. ^6^ | SOD1 (G93A) | male | SC | 80, 135 days | all immune | CD45, CD11c | FACS | sc-RNAseq |
| Butovsky, et al. ^7^ | SOD1 (G93A) | - | SC | 60 days, onset, end-stage | MG | CD39 | FACS | Nanostring nCounter |
| Butovsky, et al. ^8^ | SOD1 (G93A) | - | SC | 30, 60 days, onset, end-stage | MG | FCRLS, CD11b | FACS | Nanostring nCounter |
| Fukada, et al. ^9^ | SOD1(L126delTT) | - | SC | 98, 154, 176 days | all | - | - | microarray |
| Yoshihara, et al. ^10^ | SOD1 (G93A) | - | lumbar SC | 17 weeks | all | - | - | microarray |
| Kudo, et al. ^11^ | SOD1 (G93A) | female | lumbar SC | 8 weeks | all | - | LCM | microarray |
| Chen, et al. ^12^ | SOD1 (G93A) | female | lumbar SC | onset | all | - | - | microarray |
| Wang, et al. ^13^ | SOD1 (G93A) | female | lumbar SC | 60 days | all | - | - | microarray |
| D'Arrigo, et al. ^14^ | SOD1 (G93A) | male | lumbar SC | 55, 110 days | all | - | - | microarray |
| Baker, et al. ^15^ | SOD1 (G93A) | male | lumbar SC | 90, 120 days | ASTRO | ALDH1L1 | LCM | microarray |

**Supplementary Table 2**

**Reference gene set information summary.** A table summarizing the gene sets acquired from the reference studies that were used in the meta-analysis including essential information about samples and their processing. SC – spinal cord, ASTRO – astrocytes, OLIGO – oligodendrocytes, MG – microglia, TRAP – translating ribosome affinity purification, LCM – laser capture microdissection.

**Supplementary Table 3 (excel table)**

Marker genes of clusters of astrocytes, microglia and oligodendrocytes calculated by Seurat’s FindAllMarkers function, using Wilcoxon Rank Sum test. Default log_2_FC > 0.25 and minimal percentage of expressing cells = 0.1 filters were applied by the function. Genes are ordered based on p_adj_ value (Bonferroni correction). The most relevant marker genes can be further examined by setting a p_adj_ value threshold.

**Supplementary Table 4**

**Numerical details of individual samples.** A table summarizing numerical information about individual samples in the data set. The column ‘# Cells after all QC’ shows numbers of cells of all cell types after empty droplets, doublets and Undefined were removed and filters for number of transcripts, genes and percentage of mitochondrial reads were applied. QC – quality control, ASTRO – astrocytes, OLIGO – oligodendrocytes, MG – microglia, DEA – differential expression analysis.

|  | **Sequencing Data Details** | | | | **# Cells Entering DEA** | | | **# Cells in Subpopulation Analysis** | | |
| --- | --- | --- | --- | --- | --- | --- | --- | --- | --- | --- |
| **Sample** | **# Reads per Sample** | **% Reads Mapped to Unique Genes** | **# Cells after emptyDrops** | **# Cells after all QC** | **ASTRO** | **MG** | **OLIGO** | **ASTRO** | **MG** | **OLIGO** |
| 1M CTRL | 148890042 | 53 | 2509 | 812 | 185 | 306 | 205 | 185 | 306 | 205 |
| 1M SOD1 | 158980711 | 56 | 4394 | 2285 | 521 | 1006 | 477 | 521 | 1006 | 477 |
| 2M CTRL | 151611697 | 53 | 5484 | 3173 | 1069 | 949 | 922 | 1069 | 949 | 922 |
| 2M SOD1 | 98385405 | 50 | 5505 | 3193 | 1248 | 972 | 775 | 1248 | 972 | 775 |
| 3M CTRL | 149987478 | 55 | 5865 | 3110 | 913 | 966 | 982 | 726 | 966 | 709 |
| 3M SOD1 | 150892591 | 56 | 6172 | 3327 | 894 | 1431 | 726 | 886 | 1431 | 721 |
| 4M CTRL | 179574132 | 45 | 2693 | 1178 | 287 | 560 | 269 | 287 | 560 | 269 |
| 4M SOD1 | 207933457 | 60 | 6795 | 4502 | 370 | 2224 | 1798 | 370 | 2224 | 1798 |
|  |  |  |  |  |  |  |  |  |  |  |
|  |  | **Total** | 39417 | 21580 | 5487 | 8414 | 6154 | 5292 | 8414 | 5876 |

**
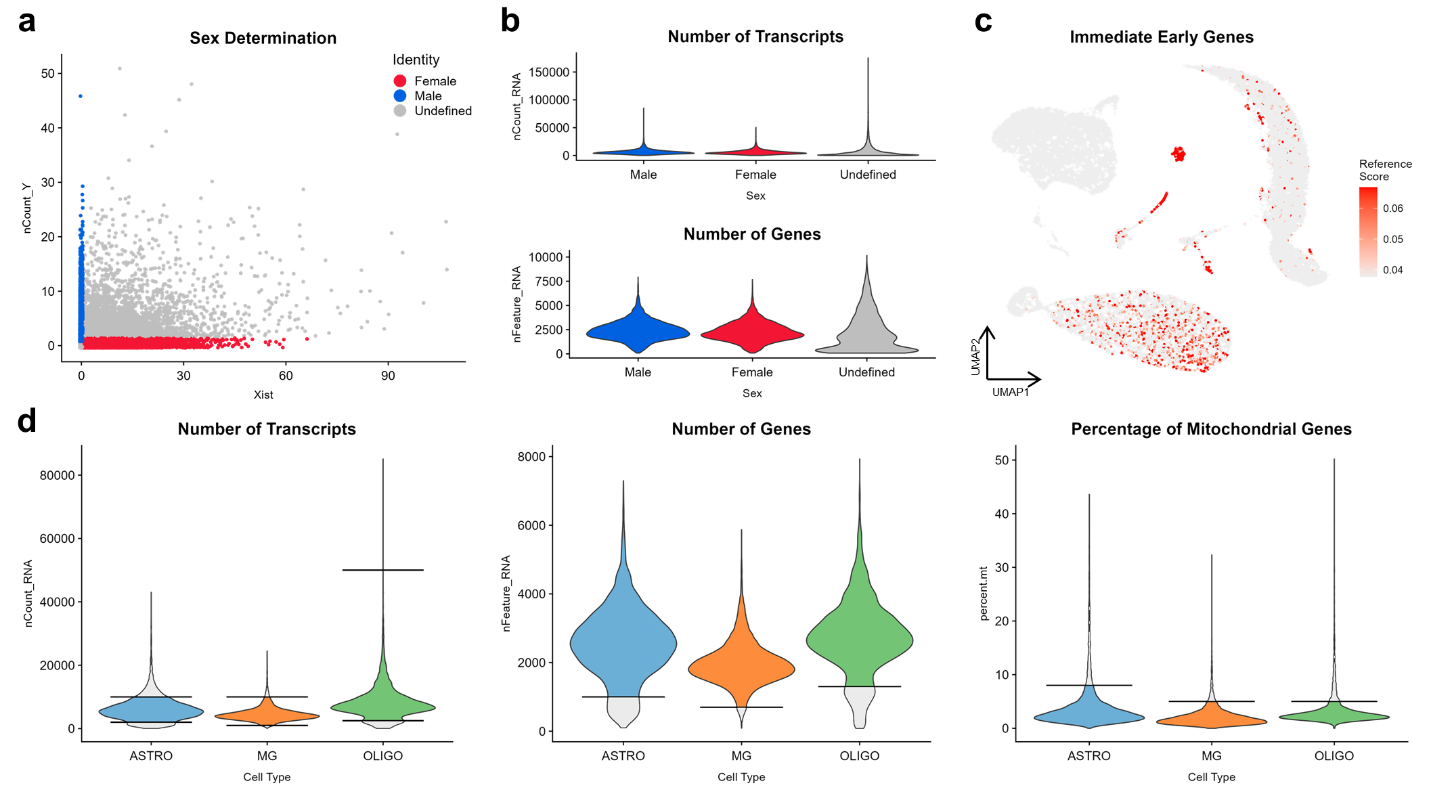
**

**Supplementary Figure 1**

**Initial quality control and filtering of the scRNA-seq data. (a)** Xist and Y chromosome gene counts of each cell visualised in a plot, with the assigned genders highlighted by colours. Undefined cells represented 29 % of all cells after removal of empty droplets and doublets. **(b)** Distribution of number of transcripts and genes detected in the Undefined group suggested it comprised of low-quality cells, therefore it was removed from the data. **(c)** A set of the immediate early genes projected onto the UMAP showing their minimal artificial activation in our data. **(d)** Plots of variables based on which the cell types of interest were filtered, with the cut-offs indicated by horizontal lines in the plots and the coloured areas representing cells that remained after filtering.


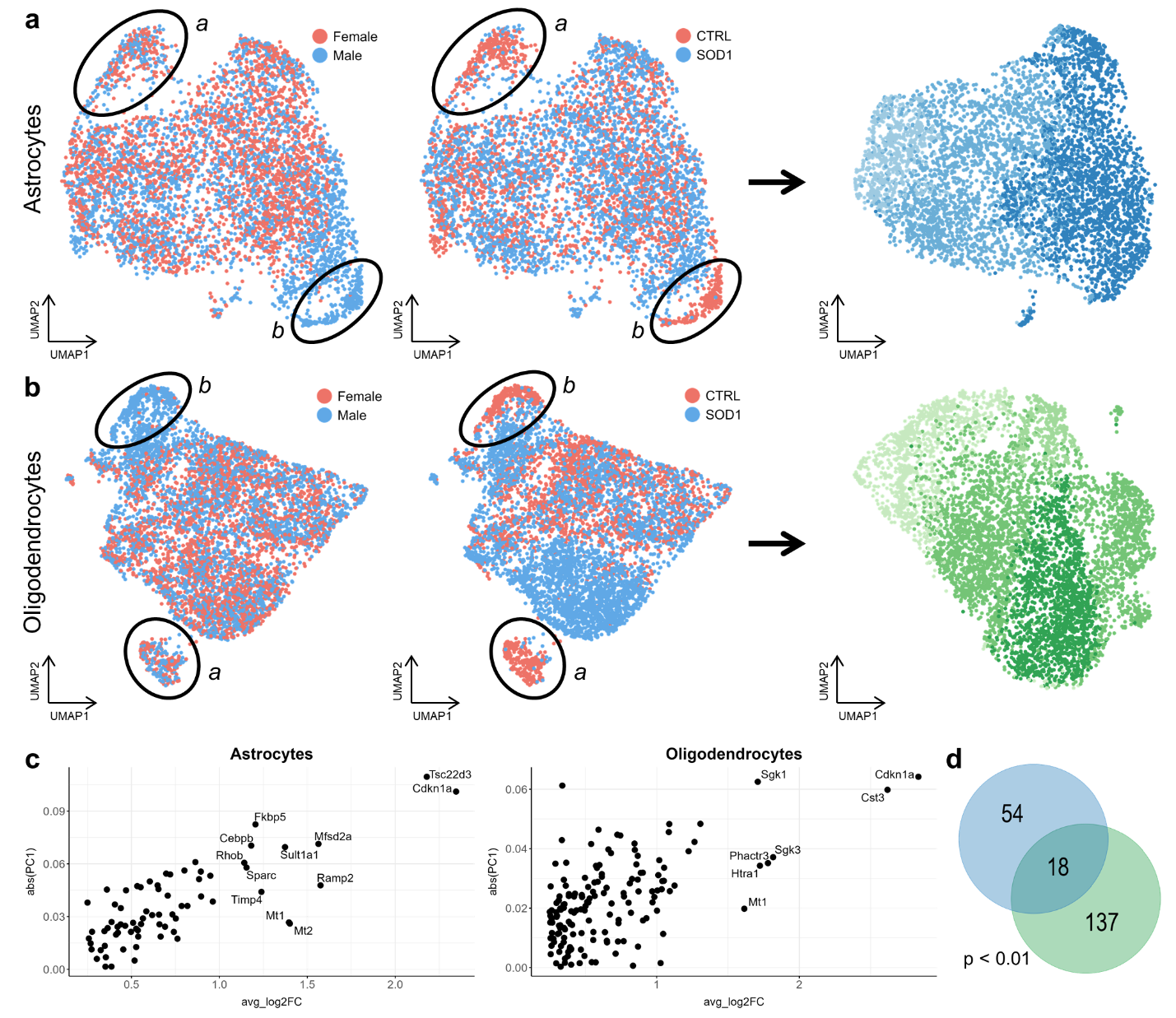


**Supplementary Figure 2**

**Subclustering quality control. (a, b)** A cluster marked by expression of *Cdk8* and *Cmss1* (*a*) included almost all 4M CTRL cells regardless of sex was present in astrocytes**,** oligodendrocytes and in microglia as well. Another cluster (*b*) was present exclusively in 3M CTRL male samples in astrocytes (n = 187) and oligodendrocytes (n = 273) but was not apparent in microglia. Exclusion of these genes and the cluster resulted in the final visualisation on the right. **(c)** Marker genes of the 3M male cluster were responsible for the shift along the PC1 axis in the pseudobulk analysis, as the strongest markers had the most extreme absolute values of loading of PC1 as shown in the plots. **(d)** Markers of this cluster in astrocytes (blue) and oligodendrocytes (green) significantly overlapped and were possibly related to stress response. abs(PC1) – absolute value of loading of PC1, avg_log2FC – log_2_FC of cluster marker genes.

**Supplementary References**

1 Ferraiuolo, L. *et al.* Dysregulation of astrocyte-motoneuron cross-talk in mutant superoxide dismutase 1-related amyotrophic lateral sclerosis. *Brain* **134**, 2627-2641, doi:10.1093/brain/awr193 (2011).

2 Sun, S. *et al.* Translational profiling identifies a cascade of damage initiated in motor neurons and spreading to glia in mutant SOD1-mediated ALS. *Proc Natl Acad Sci U S A* **112**, E6993-7002, doi:10.1073/pnas.1520639112 (2015).

3 Phatnani, H. P. *et al.* Intricate interplay between astrocytes and motor neurons in ALS. *Proc Natl Acad Sci U S A* **110**, E756-765, doi:10.1073/pnas.1222361110 (2013).

4 Miller, S. J., Glatzer, J. C., Hsieh, Y. C. & Rothstein, J. D. Cortical astroglia undergo transcriptomic dysregulation in the G93A SOD1 ALS mouse model. *J Neurogenet* **32**, 322-335, doi:10.1080/01677063.2018.1513508 (2018).

5 Liu, W. *et al.* Single-cell RNA-seq analysis of the brainstem of mutant SOD1 mice reveals perturbed cell types and pathways of amyotrophic lateral sclerosis. *Neurobiol Dis* **141**, 104877, doi:10.1016/j.nbd.2020.104877 (2020).

6 Keren-Shaul, H. *et al.* A unique microglia type associated with restricting development of Alzheimer's disease. *Cell* **169**, 1276-1290 e1217, doi:10.1016/j.cell.2017.05.018 (2017).

7 Butovsky, O. *et al.* Modulating inflammatory monocytes with a unique microRNA gene signature ameliorates murine ALS. *J Clin Invest* **122**, 3063-3087, doi:10.1172/JCI62636 (2012).

8 Butovsky, O. *et al.* Targeting miR-155 restores abnormal microglia and attenuates disease in SOD1 mice. *Ann Neurol* **77**, 75-99, doi:10.1002/ana.24304 (2015).

9 Fukada, Y. *et al.* Gene expression analysis of the murine model of amyotrophic lateral sclerosis: studies of the Leu126delTT mutation in SOD1. *Brain Res* **1160**, 1-10, doi:10.1016/j.brainres.2007.05.044 (2007).

10 Yoshihara, T. *et al.* Differential expression of inflammation- and apoptosis-related genes in spinal cords of a mutant SOD1 transgenic mouse model of familial amyotrophic lateral sclerosis. *J Neurochem* **80**, 158-167, doi:10.1046/j.0022-3042.2001.00683.x (2002).

11 Kudo, L. C. *et al.* Integrative gene-tissue microarray-based approach for identification of human disease biomarkers: application to amyotrophic lateral sclerosis. *Hum Mol Genet* **19**, 3233-3253, doi:10.1093/hmg/ddq232 (2010).

12 Chen, H. *et al.* Differential expression and alternative splicing of genes in lumbar spinal cord of an amyotrophic lateral sclerosis mouse model. *Brain Res* **1340**, 52-69, doi:10.1016/j.brainres.2010.03.075 (2010).

13 Wang, R., Yang, B. & Zhang, D. Activation of interferon signaling pathways in spinal cord astrocytes from an ALS mouse model. *Glia* **59**, 946-958, doi:10.1002/glia.21167 (2011).

14 D'Arrigo, A. *et al.* Transcriptional profiling in the lumbar spinal cord of a mouse model of amyotrophic lateral sclerosis: a role for wild-type superoxide dismutase 1 in sporadic disease? *J Mol Neurosci* **41**, 404-415, doi:10.1007/s12031-010-9332-2 (2010).

15 Baker, D. J. *et al.* Lysosomal and phagocytic activity is increased in astrocytes during disease progression in the SOD1 (G93A) mouse model of amyotrophic lateral sclerosis. *Front Cell Neurosci* **9**, 410, doi:10.3389/fncel.2015.00410 (2015).
